# Phylogenetic Analysis of Multi-Drug Resistant *Klebsiella pneumoniae* Strains From Duodenoscope Biofilm: Microbiological Surveillance and Reprocessing Improvements for Infection Prevention

**DOI:** 10.3389/fpubh.2019.00219

**Published:** 2019-08-06

**Authors:** Massimo Ciccozzi, Eleonora Cella, Alessia Lai, Lucia De Florio, Francesca Antonelli, Marta Fogolari, Francesco Maria Di Matteo, Margherita Pizzicannella, Benedetta Colombo, Giordano Dicuonzo, Silvia Angeletti

**Affiliations:** ^1^Unit of Medical Statistics and Molecular Epidemiology, University Campus Bio-Medico of Rome, Rome, Italy; ^2^Department of Biomedical and Clinical Sciences “L. Sacco,” University of Milan, Milan, Italy; ^3^Unit of Clinical Laboratory Science, University Campus Bio-Medico of Rome, Rome, Italy; ^4^Digestive Endoscopy Unit, Bio-Medico of Rome, Rome, Italy; ^5^Healthcare Associated Infection Control Committee, University Campus Bio-Medico of Rome, Rome, Italy

**Keywords:** endoscope, phylogeny, infection control, MDR *Klebsiella pneumoniae*, microbiological surveillance

## Abstract

Duodenoscopes have been described as potential vehicles of patient-to-patient transmission of multi-drug resistant organisms. Carbapenem-resistant *Enterobacteriaceae* duodenoscope related infections have been described by the Center for Disease Control and the US Food and Drug Administration consequently to outbreaks occurring in the United States. These evidences suggested that improved microbiological surveillance and endoscope design optimization could represent valid tools to improve infection control. At this aim, in this study an example of duodenoscope microbiological surveillance and reprocessing improvement analyzing strains component of bacterial biofilm by phylogenetic analysis has been proposed. From September 2016 to December 2017, duodenoscope instruments were subjected to microbial surveillance by post-reprocessing cultures of liquid collected by internal channels of instruments after injection and aspiration cycles and membrane filtration. During surveillance seventeen *Klebsiella pneumoniae*, of which 10/17 (58.8%) MDR and KPC strains were collected from duodenoscope instruments plus one MDR *Klebsiella pneumoniae* strain from the rectal swab performed before ERCP procedure in an inpatient. The surveillance allowed evidencing potential failure of reprocessing procedure and performing consequent reprocessing improvements including the contaminated instruments quarantine until their negativity. Phylogenetic analysis of whole genome sequence of duodenoscope strains plus inpatients MDR strains, showed intermixing between duodenoscopes and inpatients, as evidenced by minimum spanning tree and time-scale Maximum Clade Credibility tree. In minimum spanning tree, three groups have been evidenced. Group I including *Klebsiella pneumoniae* strains, isolated from inpatients before microbiological surveillance adoption; group II including intermixed *Klebsiella pneumoniae* strains isolated from inpatients and *Klebsiella pneumoniae* strains isolated from duedonoscopes and group III including *Klebsiella pneumoniae* strains exclusively from duedonoscope instruments. In the Maximum Credibility Tree, a statistically supported cluster including two *Klebsiella pneumoniae* strains from duedonoscope instruments and one strains isolated from an inpatient was showed. From the first microbiologic surveillance performed on September 2016 and after the reprocessing improvement adoption, none MDR or susceptible *Klebsiella pneumoniae* strain was isolated in the following surveillance periods. In conclusion, these results should encourage hospital board to perform microbiological surveillance of duodenoscopes as well as of patients, by rectal swabs culture, and rapid molecular testing for antimicrobial resistance before any endoscopic invasive procedure.

## Introduction

Multi-drug resistant organisms (MDROs) make it extremely difficult to devise a proper plan and its implementation for control (1). Guidelines for the sterilization and disinfection of invasive devices and medical instruments used for surgeries were developed, as the infection rates tend to raise (2–4). Lack of compliance with the guidelines, potentially leads to the transmission of nosocomial infections.

There is the need to develop new diagnostics and tools in healthcare institutes to contrast the evolving resistance and spread of MDROs causing healthcare associated infections (HAIs) (5). The devices used in clinical practice are considered real vehicles form MDROs transmission and HAIs spreading.

Duodenoscopes, specialized endoscopes used for endoscopic retrograde cholangiopancreatography (ERCP) have been described as potential vehicles of patient-to-patient transmission (6). Since 2014, these have been investigated for carbapenem-resistant *Enterobacteriaceae* infections by the Center for Disease Control (CDC) and the US Food and Drug Administration (FDA) consequently to outbreaks occurring across the United States (7–11).

During gastrointestinal endoscopy, endoscopes are exposed to millions of bacteria that constitute the gut flora. Unlike “critical” devices (such as laparoscopes), which require sterilization because they breach sterile tissue planes, endoscopes are currently categorized as “semicritical” devices (12), which require mechanical cleaning followed by high-level disinfection (HLD). Flexible endoscopes are semi-critical devices that cannot undergo to steam-sterilization.

In several reports, endoscope and its elevator structure have been involved in bacterial contaminations consequently to ERCP procedure. The evidence of ERCP-related infections suggested that improved microbiological surveillance and endoscope design optimization could represent valid tools to assure patient safety (8, 13–18).

The most frequent MDR pathogen involved in the described outbreaks was *Klebsiella pneumoniae* and duodenoscopes cultures were positive even after reprocessing in two-third of cases (19, 20).

It is common in hospital setting the use of gas sterilization using ethylene oxide, but this involves carcinogens and is time-consuming, expensive, and not universally available. Disconcertingly, sterilization may be ineffective if mechanical disruption of biofilm on the endoscope is incomplete.

A biofilm is an assemblage of microbial cells irreversibly attached to a surface and enclosed in a matrix of exopolymeric substances (21). A typical biofilm could contain around 85% polymeric substances and only 15% bacterial mass, with cells located in matrix-enclosed “towers” and “mushrooms” (22, 23). The ability to form biofilms allows microorganisms to survive under conditions of drying and after exposure to chemical or antimicrobial compounds (24, 25).

There is the need to eradicate the possibility of microorganisms transmission using these device, especially improving the real-time microbial biofilm detection and identification by micro-probe able to visualize the possible biofilm presence even after adequate protocol of disinfection, drying and surveillance culture. This could increase the microbial contamination assessment.

At this purpose, this study reports an example of duodenoscope microbiological surveillance and reprocessing improvement analyzing strains component of bacterial biofilm by phylogenetic analysis. After a first endoscopic microbiologic surveillance, some strains of MDR *Klebsiella pneumoniae* were isolated from duedonoscope instruments on September 2016. Phylogenetic analysis was applied to these strains to assess their origin and evolution along with other MDR *Klebsiella pneumoniae* isolates causing nosocomial infections. Based on the phylogenetic evidence, some improvements in the reprocessing protocol for the instruments used in the hospital clinical routine were decided and their efficiency checked by microbiologic surveillance from October 2016 to December 2017.

## Methods

### Operative Reprocessing Protocol in Use Until September 2016

The pre-cleaning step was performed by wiping the external part of the endoscope and flushing the internal channels with an enzymatic solution (Proteozim Plus 400, Cantel Medical).

During the manual cleaning, each internal channel was wiped and brushed with multiple-use brushes and irrigated with water and detergent solution.

The HLD phase was performed by ETD 3 Plus (Olympus) cycle 2 (detersion and disinfection without drying).

### Baseline Microbiologic Surveillance in September 2016

The surveillance was applied monthly (at least 12–24 h after the reprocessing procedure) to five duodenoscopes and three linear echoendoscopes. Instruments characteristics, and number of procedures made in the study period (1st September 2016–31st December 2017) are reported in Table 1.

**Table 1:** Features of the instruments, dates of their acquisition, and number of endoscopic procedures made in the study period.

**Manufacturer**	**Instrument type**	**Model**	**Acquisition date**	**Number of procedures from 1st September 2016–31st December 2017**
Olympus	Duodenoscope	TJF Q180V	07/15/2011	120
Olympus	Duodenoscope	TJF 145	09/27/2004	150
Olympus	Duodenoscope	TJF 145	04/09/2008	242
Olympus	Duodenoscope	TJF 160 R	10/05/2000	75
Pentax	Duodenoscope	ED-3490TK	01/19/2012	36
Pentax	Linear echoendoscope	EG-3870 UTK	05/14/2010	180
Pentax	Linear echoendoscope	EG 3870 UTK	05/04/2015	179
Pentax	Linear echoendoscope	EG-3870 UTK	07/23/2014	235

### Post-reprocessing Microbiologic Cultures

**Figure 1:**
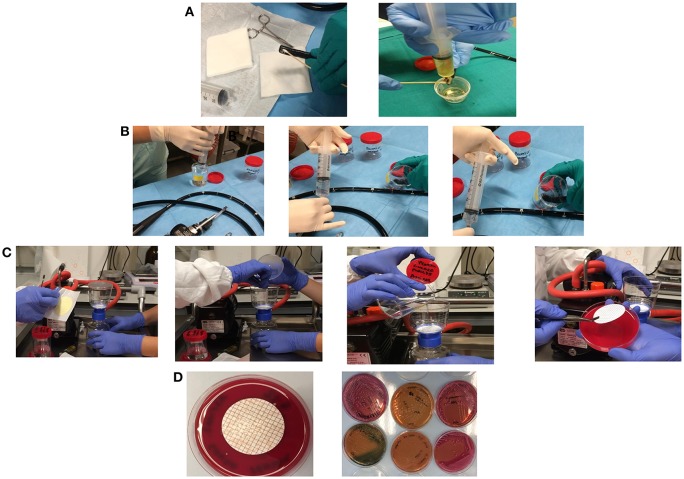
Sampling of the elevator mechanism, recess, and channel and the liquid collected in a sterile tube **(A)**. Sampling of water/air channel, biopsy channel, aspiration channel, and auxiliary channel (if there is any) by injection of sterile water holding the distal end of the endoscope within a sterile container in which gather the liquid **(B)**. Membrane filtration of the solution collected from each channel, by gridded filter placed on blood agar plates **(C)**. Colonies growing on agar plates were counted, recorded as CFU/ml, and isolated for identification **(D)**.

For elevator mechanism sampling, 20 ml of sterile water and a sterile swab were used to sample the inside recess, and channel and the liquid collected (Figure 1A). For the other endoscopes channels 20 ml of sterile water in case of the auxiliary channel (if there is any), 50 ml for the water/air channel, 50 ml for the biopsy channel and 50 ml for the aspiration channel were used. The sterile water was injected in the channel holding the distal end of the endoscope within a sterile container in which gather the liquid. Each channel was flushed with the solution from the valve to the distal end to collect sample in sterile containers. At the end, a flushing with sterile air into the channel was performed to create a mechanical turbulence within the channel allowing the potential detachment of particles in case of presence of biofilms (Figure 1B). Membrane filtration of the solution collected from each channel, by gridded filter placed on blood agar plates was performed (Figure 1C). Agar plates with filter were incubated at 35–37°C and observed after 24 and 48 h of incubation. Colonies growing were counted, recorded as CFU/ml and identified by MALDI-TOF (Bruker Daltonics, Germany) (Figure 1D). Antimicrobial susceptibility was tested by Vitek2 Compact (bioMérieux, France).

### Interpretation of Post-reprocessing Cultures and Corrective Actions

Positive cultures were classified as “low-concern” bacteria including coagulase-negative staphylococci, micrococci, diptheroids, *Bacillus* spp., and other gram-positive rods, and “high-concern” bacteria such as *Staphylococcus aureus, Enterococcus* spp., *Streptococcus* sp. viridians group, *Pseudomonas aeruginosa, Klebsiella* spp., *Salmonella* spp., *Shigella* spp. and other enteric gram-negative bacilli (7).

In case of low-concern bacteria detection at <10 CFU, reprocessing and reintroduction in the clinical procedure is suggested. If more than 10 CFU are detected reprocessing and second culture are recommended. Any number of high-concern organisms requires, reprocessing, second culture again and quarantine until the cultures are negative or below the acceptable levels of low concern organism (https://www.fda.gov/downloads/MedicalDevices/ProductsandMedicalProcedures/ReprocessingofReusableMedicalDevices/UCM597949.pdf).

### Two Step Reprocessing Improvement

The reprocessing protocol from its first version was updated with the introduction of DNP+0.5% Thiosulfate in place of the sterile water in February 2017, for its tensioactive property facilitating biofilm detection (26); the introduction of single-use brushes for the manual cleaning; the replacement of the automatic endoscope reprocessors with Soluscope series four (Soluscope, Aubagne, France); the acquisition of new cabinets DSC 8000 (Soluscope, Aubagne, France) designed to store endoscopes horizontally with continuous filtered air flow through each channels.

### Instruments Quarantine

In case of positive cultures for high-level concern microorganisms the instrument was quarantined than it was manually cleaned and automatically disinfected and cultured again until negativity.

### *Klebsiella pneumoniae* Strains Collection

Between September 2016 and December 2017, 17 *Klebsiella pneumoniae*, of which 10/17 (58.8%) MDR and KPC strains were consecutively collected from duodenoscope instruments during the microbial surveillance plus one *Klebsiella pneumoniae* MDR and KPC strain isolated from the rectal swab collected before ERCP procedure in an inpatient of the University Hospital Campus Bio-Medico of Rome, Italy. The rectal swab was cultured on selective media for Gram-negative carbapenem resistant growth and incubated at 35°C for 24 h. Suspected colonies were identified by MALDi-TOF and antimicrobial susceptibility tested as previously described (26). The 17 strains were isolated during microbiological surveillance of seven duedenoscope instruments.

### Whole-Genome Sequencing (WGS)

Bacterial DNA was extracted by the EZ1 DNA tissue kit (Qiagen, Dusseldorf, Germany) and whole genome sequenced by Next Generation Sequencing using Illumina MiSeq II sequencer (Library Preparation Kit: Nextera XT DNA Sample Prep Kit, Indexing: Dual Indexing Reagent Kits: MiSeq Reagent Kit v3, Analysis Workflow: Resequencing, Analysis Software: MiSeq Reporter). Sequencing reads from the isolates obtained in this study were assembled, after demultiplexing, single FASTQ output files of raw reads were filtered by length and quality threshold of 30. *De novo* assemblies were constructed using the SPAdes (27).

### Multilocus Sequence Typing (MLST)

MLST was performed according to the protocol described by Diancourt et al. (28) based on seven housekeeping genes: gapA (glyceraldehyde 3-phosphate dehydrogenase), infB (translation initiation factor 2), mdh (malate dehydrogenase), pgi (phosphoglucose isomerase), phoE (phosphorine E), rpoB (betasubunit of RNA polymerase), and tonB (periplasmic energy transducer). Data were pulled from WGS by submission to the MLST database used for *K. pneumoniae* genome is available at http://www.pasteur.fr.

### Phylogenetic Analysis

#### Two Datasets Have Been Created

The first dataset included 18 *Klebsiella pneumoniae* MDR and KPC strains plus 22 ST512 strains isolated from inpatients according to the ethical standards laid down in the Declaration of Helsinki, before microbiological surveillance adoption, as previously described (26). The second dataset included only *Klebsiella pneumoniae strains* ST 512 isolated from duodenoscope instruments (four strains) and *Klebsiella pneumoniae strains* ST 512 *isolated from* patients before microbiological surveillance adoption (22 strains).

*De novo* assemblies were annotated using Prokka, which relies on external feature prediction tools to identify the coordinates of genomic features within contigs (29). The pangenome was then assessed to determine the core genome, which typically includes housekeeping genes for cell envelope or regulatory functions. Pangenome analyses was performed using Roary (30).

Single-nucleotide polymorphisms (SNPs) were based on the core genome shared by all isolates. SNPs were extracted as variable sites using MEGA 7 (31) removing all ambiguous sites and gaps from the core genome alignment.

Phylogenetic signal was assessed by likelihood mapping using Tree-Puzzle (32). A transitions/transversions vs. divergence graph as well as the Xia's test of substitution saturation were implemented in DAMBE (33).

The HKY+I+G nucleotide substitution model was chosen as best-fitting model by using the hierarchical likelihood ratio test (Modeltest, implemented in PAUP^*^4). Statistical support for internal branches of the Maximum Likelihood (ML) tree was evaluated by bootstrapping (1,000 replicates) and fast likelihood-based sh-like probability (SH-aLRT). ML analysis was performed with RaxML (34) and visualized in FigTree 1.4.2.

### Minimum Spanning Tree

A minimum spanning tree (MST) using an in-house script implemented in R by the Kruskal's minimum spanning tree in boost (mstree.kruskal) was built (35, 36) on SNPs alignment (http://www.boost.org/libs/graph/doc/index.html) (first dataset).

An MST is an un-directed scheme connecting the sequences represented by all the vertices, without any cycles. Each edge is proportional to the number of SNPs separating any two vertices (sequences), by minimizing the possible total edge length (37).

### Bayesian Phylogenetic Analysis

Analysis of the temporal signal and “clocklikeness” of molecular phylogenies on the datasets was performed using TempEst (38). This analysis was performed to evaluate the robustness in terms of molecular clock of the second dataset.

Bayesian Markov Chain Monte Carlo (MCMC) method, implemented in BEAST v. 1.8.2 (http://beast.bio.ed.ac.uk) (39, 40) was used to estimate the demographic history of the *Klebsiella pneumoniae* ST512 using an evolutionary rate estimated previously (26) by calibrating a molecular clock. In order to investigate the demographic history, independent MCMC runs were carried out enforcing both a strict and relaxed clock with an uncorrelated log normal rate distribution and one of the following coalescent priors: constant population size, exponential growth, non-parametric smooth skyride plot Gaussian Markov Random Field (GMRF), and non-parametric Bayesian skyline plot (BSP) (40–42) with ascertainment bias correction. Marginal likelihoods estimate for each demographic model were obtained using path sampling and stepping stone analyses (43–45). Uncertainty in the estimates was indicated by 95% highest posterior density (95% HPD) intervals, and the best fitting model for each data set was by calculating the Bayes Factors (BF) (43, 46). In practice, any two models can be compared to evaluate the strength of evidence against the null hypothesis (*H*_0_), defined as the one with the lower marginal likelihood: 2*ln*BF <2 indicates no evidence against *H*_0_; 2–6, weak evidence; 6–10: strong evidence, and >10 very strong evidence. Chains were conducted for at least 100 × 10^6^ generations and sampled every 10,000 steps for each molecular clock model. Convergence of the MCMC was assessed by calculating the ESS for each parameter. Only parameter estimates with ESS's of >250 were accepted. The maximum clade credibility (MCC) tree was obtained from the trees posterior distributions, after a 10% burn-in, with the Tree-Annotator software v 1.8.2, included in the Beast package (39, 40). Statistical support for specific monophyletic clades was assessed by calculating the posterior probability (pp > 0.90).

## Results

### Post-reprocessing Duedonoscope Microbiological Surveillance

During the microbiological surveillance, the post-reprocessing duedonoscope sampling allowed to isolate 17 *Klebsiella pneumoniae* MDR from different instruments and one *Klebsiella pneumoniae* MDR strain in a rectal swab from an inpatient before ERCP procedure. The duodenoscopes strains were isolated from different channels of the same instrument or in different instruments as reported in Table 2.

**Table 2:** *Klebsiella pneumoniae* strains isolated during the microbiological surveillance from September 2016 to December 2017.

**Strain**	**Source of isolate**	**Channel of duodenoscope**	**Sampling date**	**MLST ST**	**Antimicrobial phenotype**
1-16	Duedonoscope A	Elevator	Sept 2016	*512*	*MDR*
11-16	Duedonoscope A	Aspiration	Sept 2016	*512*	*MDR*
5-16	Duedonoscope B	Water/Air	Sept 2016	*512*	*MDR*
83-17	Duedonoscope B	Aspiration	Mar 2017	*466*	*S*
91-17	Duedonoscope B	Auxiliary	Apr 2017	*466*	*S*
95-17	Duedonoscope B	Auxiliary	Apr 2017	*466*	*S*
97-17	Duedonoscope B	Biopsy	Apr 2017	*466*	*S*
68-16	Duedonoscope C	Aspiration	Feb 2017	*New ST3540*	*S*
72-16	Duedonoscope C	Biopsy	Feb 2017	*New ST3540*	*S*
74-17	Duedonoscope D	Water/Air	Mar 2017	*307*	*MDR*
90-17	Duedonoscope D	Biopsy	Apr 2017	*307*	*MDR*
89-17	Duedonoscope D	Auxiliary	Apr 2017	*307*	*MDR*
110-17	Duedonoscope D	Aspiration	May 2017	*307*	*MDR*
109-17	Duedonoscope E	Auxiliary	May 2017	*512*	*MDR*
101-17	Duedonoscope F	Biopsy	Apr 2017	*1519*	*MDR*
103-17	Duedonoscope F	Water/air	Apr 2017	*1519*	*MDR*
112-17	Duedonoscope G	Elevator	May 2017	*307*	*S*
46-16	Human	2016	*512*	*MDR*	

### WGS and MLST Analysis

Among the 18 *Klebsiella pneumoniae* subsp. *pneumoniae* strains analyzed with WGS, STs found were ST512 (*n* = 5, 27.8%) (four strains isolated from duedonoscopes and one from human), ST307 (*n* = 5, 27.8%), followed by ST466 (*n* = 4, 22.2%); ST1519 was found in two isolates (11.1%) (Table 1).

One new ST was identified in MLST database found in two *Klebsiella pneumoniae* subsp. *pneumoniae* strains (11.1%), this new ST has the following allelic profile for the seven loci: *gapA* 18, *infB* 22, *mdh* 56, *pgi* 61, *phoE* 234, *rpoB* 105, *tonB* 357. Searching in Pasteur *Klebsiella* locus/sequence definitions database by combinations of loci, there have been found two matches with only 4 loci (*gapA, infB, mdh, pgi*): ST2698 added in the Pasteur database in 2017 and ST3216 added in Pasteur database in 2018. Interestingly inside the same instrument, duedonoscope B, at different time different sampling collected different strains belonging at two different STs; ST512 for one strain and ST466 for four strains in different channels (Table 1). It has been assigned as new MLST ST (ST3540) by the curators of the Institute Pasteur MLST system (Paris, France). The novel allelic profile is available at http://bigsdb.pasteur.fr.

### Phylogenetic Analysis

*De novo* assemblies annotated with Prokka, identified a total of 9,314 genes divided in the following categories: 1,816 core genes (found in the 99% to 100% of the strains), 1,267 soft core genes (found in the 95–99% of the strains), 2,604 shell genes (found in the 15–95% of the strains), and 3,627 cloud genes (found in the 0–15% of the strains).

**Figure 2:**
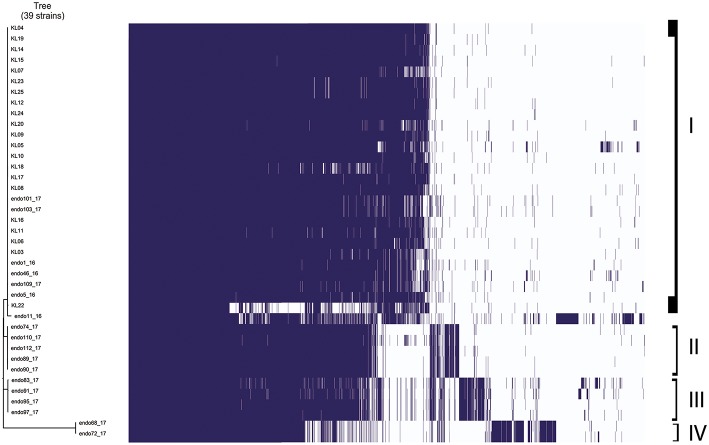
Genetic expression of *Klebsiella pneumoniae* isolates. Group I-IV are highlighted. Blue boxes represent the presence of gene expression. The white color corresponds to the “zero” value indicating the absence of over/under gene expression.

Gene absence and presence of *Klebsiella pneumoniae* strains analyzed is showed in Figure 2. It is possible to highlight four different groups based on the more similar gene presence composition: the first group included 26 *Klebsiella pneumoniae* strains in total, 22 strains isolated from inpatients plus 4 strains isolated from duedonoscopes. Between the first and second group, there were two *Klebsiella pneumoniae* strains that is possible to consider as outgroup of the first group. These strains were isolated one from inpatient and one from duedonoscopes showing an assorted gene variant composition. The second and third group included five and four *Klebsiella pneumoniae* strains isolated both from duedonoscopes, belonging to ST307 and ST406, respectively. The more divergent group in terms of presence/absence gene composition was the fifth group composed by two *Klebsiella pneumoniae* isolates, belonging to the new ST3540, isolated from two different channels of the same duedonoscope (Duedonoscope C).

Likelihood mapping analysis indicated 14.5 and 30.1% of star-like signal (phylogenetic noise) and 78.8 and 66.9% of network-like signal (Phylogenetic signal) for the first and second dataset, respectively; this indicated that enough signal for phylogenetic inference was present.

### Minimum Spanning Tree (MST) Analysis

MST is a clustering method allowing to explore potential relationships among closely related strains, assuming that in an outbreak, a chain of transmission can be represented by a graph connecting all strains with the minimum genetic distance among them.

**Figure 3:**
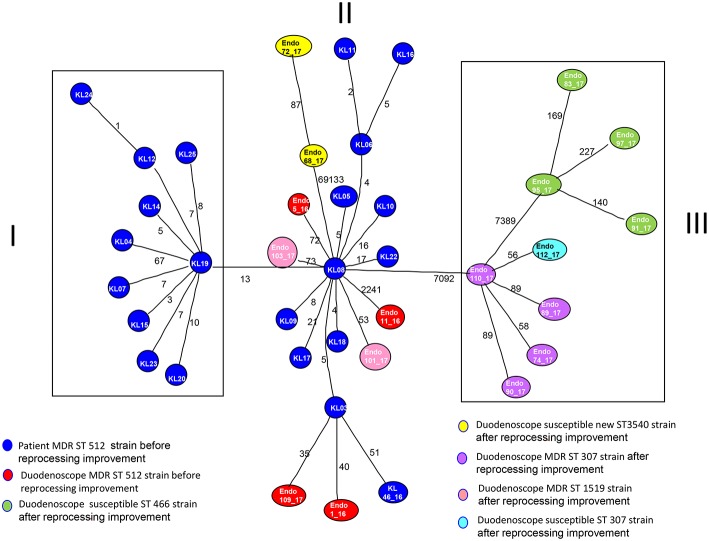
Minimum spanning tree (MST) by SNPs alignment of 22 *Klebsiella pneumoniae* MDR ST 512 strains isolated from inpatients (blue circles) plus 17 *Klebsiella pneumoniae* strains isolated from duodenoscopes. Duodenoscopes strains are indicated with circles of different colors depending on ST and antimicrobial susceptibility, according to the legend in the figure. The number along an edge is the number of SNPs separating connected sequences.

MST tree, in Figure 3, showed three internal nodes dividing MST tree in three groups, connected by two bridges of different length corresponding to 13 and 7092 SNPs numbers, respectively. Group I included 10 *Klebsiella pneumoniae* strains, isolated from inpatients in the years 2012 and 2013 before microbiological surveillance adoption, separated by at least three SNPs difference. The internal group, group II, included 12 MDR *Klebsiella pneumoniae* strains isolated from inpatients in the years 2012 and 2013 before microbiological surveillance and eight *Klebsiella pneumoniae* strains, 6 MDR strains and two susceptible strains, isolated from duedonoscopes (instrument A, B, C, E, and F). Strains belonging to group II showed intermixing between these two different sources. The internal node of this group is a MDR strain (KL-08) isolated from an inpatient before microbiological surveillance; this strain shared maximum 21 SNPs difference with the other inpatient strains, isolated before microbiological surveillance, and minimum 53 SNPs difference with the duedonoscopes strains. Patients strains (blue circles, Figure 3) were all MDR ST 512 *Klebsiella pneumoniae*, as well as four (red circles, Figure 3) strains isolated from different instruments (two from duodenoscope A, one from duodenoscope B, and one from duodenoscope D) collected in different channels (elevator, auxiliary, aspiration, and water/air) and different sampling periods (three isolates in September 2016 and one in May 2017) (Table 2)*. Two* MDR strains belonging to group II (pink circles, Figure 3) identified as ST 1,519 were collected from different channels (biopsy and water/air) of the same duodenoscope (Instrument F) in the same period (April 2017) (Table 2). They showed a low number of SNPs difference, ranging from 53 to 73, with the central strain KL-08 characterized as MDR and ST 512 strain. Noteworthy, two isolates of the group II (yellow circles, Figure 3) collected from duodenoscope C sampled in February 2017 (Table 2), one from the aspiration channel and the other from the biopsy channel, were assigned as new MLST ST (ST3540). These strains showed a high number (69133) of SNPs difference from the central strain KL-08 confirming the genetic distance of the new ST from the more common ST 512.

The group III included nine *Klebsiella pneumoniae* strains isolated from three duedonoscope instruments, showing a high number of SNPs difference (7092) from strain KL-08 of group II; this is in agreement with the presence in the group III, of different MLST STs (ST466 and ST307). *Klebsiella pneumoniae* ST466 strains (green circle; Figure 3) were all not resistant strains isolated from the same instrument (Instrument B) in different sampling periods (March-April 2017) and channels (one from aspiration, two from auxiliary and one from biopsy channel) (Table 2). These strains showed a high number of SNPs difference (7389) from strain 110-17 (ST 307 MDR strain) collected from the aspiration channel of a different duodenoscope (instrument D) in a different period (May 2017). Strain 110-17 showed a low number of SNPS difference, ranging from 56 to 98, from other MDR ST307 strains collected from the same instrument (duodenoscope D) in different channels (aspiration, auxiliary, biopsy, and water/air channels) and sampling period (April–May 2017) and from strain 112–17 a susceptible strain isolated in a different instrument (duodenoscope G) in May 2017 (Table 2).

### Bayesian Phylogenetic Analysis

For Bayesian phylogenetic analysis, only *Klebsiella pneumoniae* MDR ST512 strains (second dataset) have been considered with the aim to investigate the possible relationship between duedonoscopes and inpatient MDR strains.

Analysis of the temporal signal and “clock-likeness” of molecular phylogenies was performed on the second dataset. A strong correlation between the genetic distance of each sequence to the root of the Klebsiella ST512 strains SNPs phylogeny and the date of sequence sampling for the second dataset (*r* = 0.73) was found.

The Bayesian skyline plot as demographic model with a relaxed molecular clock was selected as the most appropriate to describe the evolutionary history of *Klebsiella pneumoniae* SNPs alignment (lnBF> 8). The substitution rate used for the analysis was 4.97 × 10^−3^ substitutions site per year (95% HPD 9.98 × 10^−3^- 9.67 × 10^−4^) (26).

**Figure 4:**
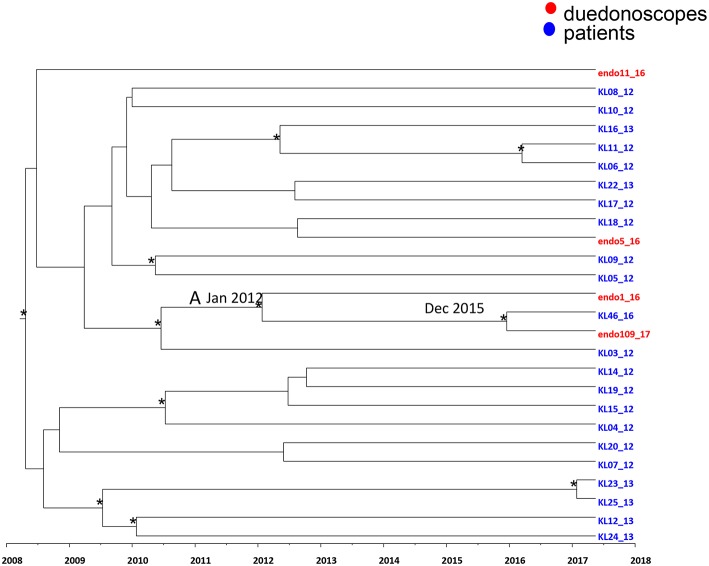
Maximum clade credibility (MCC) tree with Bayesian dated reconstruction of *Klebsiella pneumoniae* MDR ST 512 strains isolated from inpatients (blue) and duodenoscope (red). Branches are scaled in time and tips colored according to the legend to the right corner. Significant posterior probability support (pp ≥ 0.9) as indicated by an asterisk. The statistical significant clade A contains intermixing between inpatients and duodenoscope strains.

Maximum clade credibility (MCC) tree of *Klebsiella pneumoniae* ST512 SNPs alignment is showed in Figure 4.

Focusing in the MCC tree the relationship between duedonoscopes and inpatient strains, a statistically supported cluster was showed (cluster A) (Figure 4).

Cluster A included three *Klebsiella pneumoniae* strains: two strains were isolated from duedonoscopes (endo 1-16 and endo 109-17) sampled in 2016 and 2017, respectively, plus one strain isolated in one inpatient (KL 46-16) in the year 2016. The date of the time of the most common recent ancestor (tMRCA) of the cluster A corresponded to January 2012. The inpatient strain clustering together with the duedonoscope strain isolated in 2017 dated back to December 2015.

The inpatient strain (KL46-16) was isolated in a rectal swab at the admission in the hospital before the patient underwent to ERCP. For this procedure, a duedonoscope included in the microbiological surveillance (duedonoscope E) was used. The duedonoscope strain (endo109-17) was isolated in 2017 from the duedonoscope E used for ERCP in the patient KL46-16.

The other two *Klebsiella pneumoniae* strains isolated from duedonoscopes were interspersed within the tree clustering with other inpatient *Klebsiella pneumoniae* strains these clusters were not statistically supported.

### Two Step Reprocessing Improvement

From the first microbiologic surveillance performed on September 2016 and during the reprocessing improvement period, a significant number 10/17 (66.7%) of MDR gram-negative strains have been identified in different duodenoscopes surveilled, as reported in Table 2. MDR Gram-negative strains were identified (Figure 1D). The introduction of thiosulfate in place of the sterile water, determined a relative increase in detection of gram-negative pathogens during the surveillance period until May 2017. In case of positive cultures for high-level concern microorganisms, the instruments were quarantined, reprocessed and sampled again for cultures, until negativity. During this period, the positive instruments were kept in quarantine and excluded from the clinical routine.

**Figure 5:**
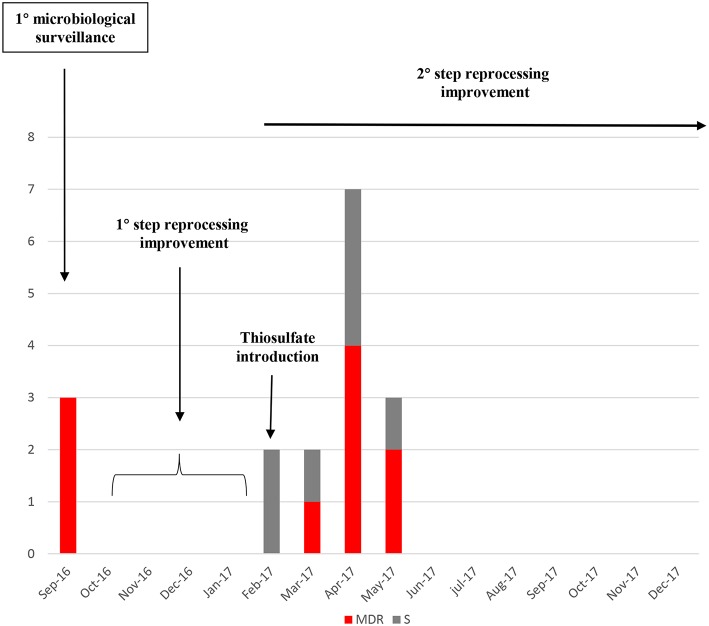
Number of *Klebsiella pneumoniae* strains isolated from the start of the microbiological surveillance and during the reprocessing improvement of the duodenoscopes. MDR strains are evidenced in red, susceptible strains in gray.

After the reprocessing improvement and parallel preventive quarantine of contaminated duodenoscopes, none MDR or susceptible *Klebsiella pneumoniae* strain was isolated in the following surveillance periods (Figure 5).

## Discussion

During the endoscopic procedure, the surface of the instrument as well as the internal channel are exposed to fluids and microorganisms potentially infectious. Flexible endoscopes are not adequate for steam sterilization, consequently the reprocessing procedure can be achieved by detergent cleaning and high-level disinfection. Since 2012, patient-to-patient transmission of MDR microorganisms, such as carbapenem-resistant *Enterobacteriaceae*, has been supposed in many reports and potentially ascribed to the persistent contamination of the elevator mechanism even in case of apparent respect of the reprocessing procedure (6, 13). Critical steps are represented by inadequate manual cleaning, brushing, channel flushing, and insufficient drying before storage (47).

In addition, the characteristic moist environment into the flexible endoscopes, facilitate the bacterial growth promoting the ability of infection transmission if the reprocessing is not completely achieved.

The evidence of ERCP-related infections suggested that the microbiological surveillance is essential and could represent a valid tool limiting these infections and assuring patient safety (6, 8, 13–18).

In this study, the analysis of different *Klebsiella pneumoniae* strains isolated from duedonoscopes as well as from inpatients infections highlighted a patchwork of different strains. The most prevalent STs were represented by ST512 and ST307 in 27.8% of cases, followed by ST466 and ST1519. Interestingly, two strains belonging to a new ST (ST3540), suggesting how these invasive devices could be real transmission vehicles. It is conceivable that the different microorganisms forming a biofilm within the duodenoscope surface could survive under conditions of drying and chemical or antibiotic exposure and mutate to this scope.

This result is confirmed by the annotation analysis, indicating a heterogeneity even within the same ST and conversely a similarity between *Klebsiella pneumoniae* strains isolated from duedonoscope instruments and inpatient strains.

At the MST analysis, a clear separation of *Klebsiella pneumoniae* strains into three different groups was showed. Two distinct groups (group I and III) included *Klebsiella pneumonaie* strains isolated only from inpatient or duedonoscope, meanwhile an intermixed group, the group II, included strains from both origins. This result suggests a possible bi-directional way of transmission between duodenoscopes and patients showing an exchange and a relationship among them. This bidirectional way of transmission could be stopped by reprocessing improvements and parallel microbiological surveillance of duodenoscopes involved in clinical routine. When an instrument resulted positive for bacterial contamination, it was quarantined, reprocessed, and re-sampled until negativity, thus guaranteeing no further strain circulation between patient and instruments during the clinical procedure. This preventive measure allowed to control the spread of strains, even those of new introduction as for example the ST never described before, discovered in this study and classified by the Pasteur Institute as ST 3540, identified in duodenoscope C during surveillance.

At the MST tree, strains heterogeneity inside the same instruments between different channels as well as within the same channel was evidenced. Different strains of microorganisms could colonize duedonoscope instruments creating polimicrobial infections as suggested from the MLST and MST analysis if we consider also ST heterogeneity inside the instrument B. This could be due to multiple strains composition of the biofilm or to multiple strains colonization within the same channel or to a single microorganism mutating during the time.

Time-scale MCC tree, showed a statistically supported cluster including two *Klebsiella pneumoniae* strains from duedonoscope instruments and one strains isolated from an inpatient. The inpatient strain was isolated in rectal swab before the ERCP procedure, whereas the duedonoscope strain was isolated from instrument E, the instrument used for the patient ERCP procedure. The probable tMRCA of this cluster was December 2015 in accordance with the probable transmission between patient and instrument happened in 2016, during the ERCP procedure. The duodenoscope contamination time was reveled in 2017 when it was surveilled. From this positivity, the instrument quarantine protected from further transmission to other patients.

The results of this study further confirmed what it has been previously supposed that duodenoscopes, used for (ERCP) could be potential vehicles of patient-to-patient multi-drug resistant strains transmission (6). Phylogenetic analysis represents a valid tool accurately identifying this transmission chain as previously described in nosocomial setting infections (26, 48). Phylogenetic approach represents a point of strength of this study because the limited period of observation could have been not enough to evidence way of transmission of bacteria from instrument to patient and vice versa by classical epidemiology (49).

Efficacy of the reprocessing improvement and parallel microbiological surveillance of operative duodenoscope used in clinical routine was proved by the evidence that none MDR strain was isolated in the following surveillance periods.

## Conclusion

The results of the present study should encourage hospital board to perform microbiological surveillance of duodenoscopes as well as of patients by rectal swabs culture and rapid molecular testing for antimicrobial resistance before any endoscopic invasive procedure.

The microbiological surveillance of the duodenoscopes used in clinical practice, the new duodenoscope design to make easier and effective the reprocessing procedure, the real-time molecular epidemiology are all necessary steps to be improved in hospital settings for a routinely microbiological surveillance essential for patient safety during invasive procedures (50, 51).

## Data Availability

The datasets generated for this study can be found in the Institute Pasteur MLST system (Paris, France) at http://bigsdb.pasteur.it.

## Ethics Statement

Ethical Committee University Campus Bio-Medico of Rome.

## Author Contributions

MC, SA, GD, and FD conceived and designed the study. LD, FA, MF, MP, and BC collected the samples 28 and performed the experiments. EC, AL, MC, and SA analyzed the data and performed phylogenetic analysis. MC and SA wrote the paper. All authors read, reviewed, and approved the final manuscript.

### Conflict of Interest Statement

The authors declare that the research was conducted in the absence of any commercial or financial relationships that could be construed as a potential conflict of interest.
